# Contrasting group analysis of Brazilian students with dyslexia and good readers using the computerized reading and writing assessment battery “BALE”

**DOI:** 10.3389/fpsyg.2014.00837

**Published:** 2014-07-31

**Authors:** Carolina M. J. Toledo Piza, Elizeu C. de Macedo, Monica C. Miranda, Orlando F. A. Bueno

**Affiliations:** ^1^Psychobiology Department, Universidade Federal de São PauloSão Paulo, Brazil; ^2^Psychology Department, Universidade Presbiteriana MackenzieSão Paulo, Brazil

**Keywords:** dyslexia, reading skills, phonological processing, visual coding, assessment

## Abstract

The analysis of cognitive processes underpinning reading and writing skills may help to distinguish different reading ability profiles. The present study used a Brazilian reading and writing battery to compare performance of students with dyslexia with two individually matched control groups: one contrasting on reading competence but not age and the other group contrasting on age but not reading competence. Participants were 28 individuals with dyslexia (19 boys) with a mean age of 9.82 (SD ± 1.44) drawn from public and private schools. These were matched to: (1) an age control group (AC) of 26 good readers with a mean age of 9.77 (SD ± 1.44) matched by age, sex, years of schooling, and type of school; (2) reading control group (RC) of 28 younger controls with a mean age of 7.82 (SD ± 1.06) matched by sex, type of school, and reading level. All groups were tested on four tasks from the Brazilian Reading and Writing Assessment battery (“*BALE*”): Written Sentence Comprehension Test (WSCT); Spoken Sentence Comprehension Test (OSCT); Picture-Print Writing Test (PPWT 1.1-Writing); and the Reading Competence Test (RCT). These tasks evaluate reading and listening comprehension for sentences, spelling, and reading isolated words and pseudowords (non-words). The dyslexia group scored lower and took longer to complete tasks than the AC group. Compared with the RC group, there were no differences in total scores on reading or oral comprehension tasks. However, dyslexics presented slower reading speeds, longer completion times, and lower scores on spelling tasks, even compared with younger controls. Analysis of types of errors on word and pseudoword reading items showed students with dyslexia scoring lower for pseudoword reading than the other two groups. These findings suggest that the dyslexics overall scores were similar to those of younger readers. However, specific phonological and visual decoding deficits showed that the two groups differ in terms of underpinning reading strategies.

## INTRODUCTION

Developmental dyslexia is characterized by “difficulties with accurate and/or fluent word recognition and by poor spelling and decoding abilities. These difficulties typically result from a deficit in the phonological component of language that is often unexpected in relation to other cognitive abilities and the provision of effective classroom instruction” (Lyon et al., 2003). Several studies have shown there are major educational and psychosocial impacts in the lives of individuals with dyslexia, which underlines the need for well-trained healthcare professionals and educators (Bishop and Snowling, 2002; Capovilla and Capovilla, 2002; Schoen-Ferreira et al., 2002; Franco de Lima et al., 2006; Capellini et al., 2007; Fletcher, 2009).

One of the basic assumptions of a cognitive neuropsychological approach is that cognitive systems operate through relatively independent modules (Luria, 1981; Salles et al., 2004), which are also organized as systems; therefore some processes may be impaired while others remain intact. A module may be damaged without affecting another’s functionality. An example of this would be cases in which certain reading processes are adequate while others are deficient. Reading of high-frequency words might be adequate, while the reading of pseudowords or irregular words may be affected, as is often the case for reading disorders (Sternberg, 2000; Capovilla and Capovilla, 2002; Salles et al., 2004). Therefore, when assessing reading skills, it is extremely important to detect not only ability or inability to decode stimuli but also the underpinning cognitive skills related to this process (Manis et al., 1996; Bishop and Snowling, 2002; Griffiths and Snowling, 2002; Salles et al., 2004; Salles and Parente, 2006). This helps us discriminate early-stage readers from those considered “competent,” and also allows us to determine whether a child is lagging in relation to the expected level (Grégoire, 1997; Orsati et al., 2005; Salles and Parente, 2006).

Studies in regular languages such as Portuguese describe that the main difficulty observed in dyslexic individuals relates to the phonological processing, expressed by their poor performance on phonological awareness tasks, phonological working memory, and rapid automatic naming tasks. This confirms the hypothesis that one of the major causes of developmental dyslexia is a phonological deficit (Frith, 1997; Stanovich et al., 1997; Ramus et al., 2003; Shaywitz and Shaywitz, 2005; Capellini et al., 2007). Evidence supporting this hypothesis is clear in studies with dyslexic adults who have coped with initial difficulties and succeeded in reading within normal parameters, while continuing to experience phonological processing difficulties (Ramus et al., 2003; Bishop and Snowling, 2002).

In order to better identify reading and writing profiles in dyslexic individuals, Bryant and Impey (1986), over 30 years ago, started to compare children with dyslexia to two control groups – one matched by age and the other by reading level. This was done, since they assumed that reading performance depends on overall word recognition, skill which differs greatly between students with dyslexia and same-aged competent readers, for whom they are not affected. A comparison between children with dyslexia and age-matched controls may thus produce biased results, since the group with dyslexia, according to these researchers, behaves similarly to younger readers.

This innovative approach was very controversial at the time, since matching groups by reading level, differs from matching them by age (Manis et al., 1996). Criticisms were raised in relation to instruments used to assess the children’s reading level, since the approach method used to match the two groups would surely affect findings. This issue was discussed for a few years but no definitive conclusion was reached (Manis et al., 1996; Stanovich et al., 1997; Griffiths and Snowling, 2002). However, current studies continue to match groups for both reading level and age (Stanovich et al., 1997; Salles et al., 2004; Salles and Parente, 2006, 2008; Affonso et al., 2011).

Brazilian researchers (Salles et al., 2004; Salles and Parente, 2006) used the contrasting group method to compare children with reading and writing difficulties to two control groups. One of their studies showed that when individuals with dyslexia where compared to younger children, their specific difficulties were more accurately observed than when using same-age controls, who scored higher on almost all items (Salles and Parente, 2006).

These findings confirm that when investigating reading and writing processes, one must consider the psycholinguistic characteristics of the code, since regularity, frequency and type of word used (word vs. pseudoword), highly influences any investigation of such processes. Adding to that, the acquisition of literacy evolves several stages, until we reach the point at which an individual is fully literate. Therefore, our understanding of reading and writing acquisition processes in different cultures is extremely important, due to the specificity of language and also, because much of the knowledge produced by a society is transmitted in writing. (Ellis, 1995; Lecours and Parente, 1997).

The Brazilian-Portuguese computerized reading and writing assessment battery (local acronym BALE, from the original name “Bateria de Avaliação de Leitura e Escrita – Computadorizada”), developed by Macedo et al. (2002, 2005) consists of seven tasks, that assess oral language, reading and writing components, such as reading and writing of words and pseudowords, comprehension of written sentences, the latter being contrasted with comprehension on spoken (oral) sentences. As a psychometric test, its normative data enable researchers to assess the extent of deviation from reading and writing benchmarks for each educational level.

The instrument is based on principles of cognitive psychology and on the information processing theory and takes into account previous criticisms regarding reading and writing assessments that are restricted to mean scores. BALE has gained recognition due to its discriminatory capacity, as an instrument that maps individual profiles, considering the various subcomponents that comprise written language. The Reading Competence Test – (RCT; Macedo et al., 2005), is one of the BALE tasks used in the present study, that adopts this precise type of analytical procedure, enabling clinicians and researchers to assess reading strategies and interpret the nature of underlying cognitive processes being used. The computerized version of this instrument was developed to assess the reading and writing developmental stage reached by school students, while also helping to show which reading routes (phonological or lexical) and reading strategies (logographic, alphabetic, or orthographic) are predominant (Macedo et al., 2005; Capovilla et al., 2006; Teixeira et al., 2010).

Teixeira et al. (2010) administered the computerized BALE in students (with mean age 11.4) from a remedial class in an elementary education public school, and then compared these results with teachers’ qualitative assessments of their writing levels. The computerized BALE assessment provided a more sensitive profile of the persistent reading and writing difficulties, since students continued to make predominant use of the phonological route for reading and writing. Teachers’ qualitative evaluations did not fully examine underlying processes, therefore claimed they showed progress. As a result, all students automatically went on to the next grade despite not having reached a minimally acceptable level of reading.

Previous studies of students with dyslexia using the computerized BALE (Capovilla et al., 2004, 2006; Lukasova et al., 2008) compared these individuals with control group matched by age and school grade. Capovilla et al. (2004) found that students with dyslexia performed poorly on the RCT, particularly on pseudo-homophone items (pseudowords with orthographic errors but correct phoneme–grapheme conversion) and on phonologically similar words. These findings were explained by the phonological deficit theory. Lukasova et al. (2008), in another study, showed that students with dyslexia took longer to complete word reading and sentence comprehension tasks while there were no significant differences in overall reading score or oral sentence comprehension. The study concluded that if this group is given a longer period to read, they have a good chance of improving their performance.

In a more recent study, Affonso et al. (2011) used the contrasting group method to evaluate students with dyslexia and controls by administering the Picture-Print Writing Test (PPWT), another BALE task, that assesses spelling (writing) of single words. Their findings demonstrated that the dyslexia group and reading controls did not differ in the total number of correct responses. However, both groups scored below the age-matched controls. On analyzing types of errors, there were significantly different inter-group patterns: the dyslexia group made more mistakes, related to phonological skills and to a lack of automation of the orthographic process.

In general, studies applying the computerized BALE to clinical samples have confirmed that this battery is a sensitive means of investigating the cognitive processes underpinning reading and writing. It is also an efficient instrument for the differential diagnosis of learning disorders. To our knowledge, however, only Affonso’s study used one task of the computerized BALE with a contrasting group method. Therefore, the present study objectives to compare the performance of children with dyslexia with two control groups: one group matched by chronological age and the other consisting of younger control children matched by their reading level, in four tasks of the computerized BALE.

Our study also aims to investigate the instrument’s sensitivity for early identification of predominant reading strategies, which certainly enables a more specific intervention work.

## MATERIALS AND METHODS

### PARTICIPANTS

This study’s methodological design analyzed contrasting groups and compared a group of children with dyslexia with two control groups: one group matched for chronological age and the other consisting of younger controls matched for their sentence reading levels.

#### Dyslexic group (DX)

The group consisted of 28 dyslexic children of both sexes, aged 8–14, taken from public and private schools in the State of São Paulo. Of this total, 19 (67.9%) were boys with a mean age of 9.82 (±1:44) (minimum 8.0, maximum 13.8). Those attending public schools also numbered 19 (67.9%). All participants were referred for neuropsychological assessment from outpatient clinics of the Interdisciplinary Neuropsychological Child Care Group Center (local acronym NANI), between 2006 and 2008. Diagnostics were based on DSM IV-TR (American Psychiatric Association, 1995) and CID-10 criteria (for further details see Barbosa et al., 2009) and all subjects agreed to participate in the current study that was submitted and approved by the UNIFESP Research Ethics Committee (ref. 1498/07).

The following exclusion criteria were applied (1) comorbid patients with Attention Deficit Hyperactivity Disorder; (2) Total IQ scaled score (measured by WISC III) under 80; (3) less than one year age-grade discrepancy on the reading section of a Brazilian school performance test (Teste de Desempenho Escolar – (TDE; Stein, 1994); (4) uncorrected visual/auditory deficits suspected; (5) children with brain injuries or a clinical history of neurological or psychiatric illnesses; (6) delayed neuropsychomotor development and intellectual deficit.

Control groups were selected from the normatization database for the computerized BALE (Macedo et al., 2002; Nikaedo et al., 2007). Boys and girls from private and public schools, grades 1–7, were selected for these groups:

#### Age control group (AC)

It consisted of 26 children with reading levels within the expected range, based on the average for their grade, matched with dyslexic children by sex, age, and type of school. There were 17 boys (65.4%), mean age 9.77 (±1:37), all from public system schools.

#### Reading group control (RC)

It consisted of 28 younger readers matched with students with dyslexia by sex, type of school, and reading level. Reading level was scored by the number of total correct responses (maximum 40 points) on the Written Sentence Comprehension Test from the BALE computerized (task described in the next section). This group’s mean age was 7.82 (±1.06) and children from public system schools numbered 19 (67.9%).

### PROCEDURES

In order to analyze the performance of students with dyslexia and controls, four tasks of the computerized BALE were used (Macedo et al., 2002):

– Written Sentences Comprehension Test (WSCT, original name and acronym is Teste de Competência de Sentença Escrita – (TCSE; Macedo et al., 2005): a computerized test that assesses the extraction of meaning from written sentences with different difficulty levels. The test is made up of 46 items, being the first six examples. Each item is composed of six pictures, of which only one precisely represents the sentence written above it. In each item, the subject should read the written sentence and click on the picture that best corresponds to the sentence.

This instrument was used to match the dyslexic group with controls by reading level.

– Oral Sentences Comprehension Test (OSCT, original name and acronym is Teste de Competência de Sentença Falada – (TCSF; Macedo et al., 2005): is a computerized test that assesses the extraction of meaning from spoken (oral) sentences with different difficulty levels. The test consists of 46 items, each containing six pictures and one spoken sentence (by a female audio in the computer), and only one picture precisely matches the sentence. The subject must click on the picture that best describes the sentence heard. This complementary test uses the same stimuli as the WSCT.
– Reading Competence Test (RCT, original name and acronym is Teste de Competência de Leitura de Palavra e Pseudopalavra – (TCLPP; Macedo et al., 2005): a computerized test for word recognition skills and strategies by judging correspondence between picture and written word pairs. Subjects must press a “correct” or “incorrect” button according to pairs displayed. Seventy-eight items are divided into seven word-picture categories as follows: regular correct words (RC): where regular word corresponds to paired image; irregular correct words (IC): where irregular word corresponds to paired image; semantic confusion (SC): when the word is not semantically related to the picture; pseudoword with visual confusion (VC); pseudoword with phonological confusion (PhC); pseudoword with orthographic errors but correct phoneme-grapheme conversion (PNw) and pseudoword not derived from a real word (WNw).
– The Picture-Print Writing Test (PPWT, original name and acronym is Teste de Nomeação de Figura por Escrita – TENOFE)

It consists of a set of 36 items, each of which contains individual pictures to be named and spelled in writing. Pictures are centered on the screen with a blank line below for the subject to write in the words corresponding to each picture. For the present analysis, one point was attributed to each picture written correctly.

### STATISTICAL ANALYSIS

SPSS, version 13.0 was used to analyze data. Since the data were not normally distributed nor variance homogenous, inter-group comparisons were made using the Kruskal–Wallis non-parametric test followed by the Mann–Whitney test.

Mean correct responses and mean time for task completion of the computerized BALE were analyzed. Due to the fact that the RCT task enabled us to identify the individual reading levels, as well as predominant route used, the seven subtypes of response assessed in this task were analyzed separately. The level of significance was set at 5% for all tests.

## RESULTS

### OVERALL ANALYSIS OF BALE TASKS

**Table 1** shows the overall performance on the four BALE tasks, of the three groups. Picture-Print Writing Test completion time was not included in the analysis because the duration of the test also depends on the subject’s level of familiarity with computers.

**Table 1:** Mean correct responses and mean completion time per BALE task, for each of the three groups.

	DX	RC	AC	
	
	Mean (SD)	Mean (SD)	Mean (SD)	*p*-Value
WSCT correct	23.89 (±12.10)^A^	24.14 (±12.10)^A^	37.84 (1.49)^B^	0.001
OSCT correct	35.79 (±3.01)^A^	33.94 (±4.31)^A^	38.87 (±0.88)^B^	0.001
RCT correct	53.07 (±6.09)^A^	55.18 (±7.20)^A^	64.31 (±2.96)^B^	0.001
PPWT correct	15.27 (6.45)^A^	19.53 (±9.68)^B^	28.57 (±3.29)^C^	0.001
WSCT time	29335.99 (±17934.61)^A^	17301.72 (±8758.06)^B^	12094.68 (±3036.01)^C^	0.001
OSCT time	8275.74 (±2144.58)^A^	7614.66 (±2052.61)^A^	6683.25 (1330.02)^B^	0.01
RCT time	8584.68 (±4635.28)^A^	6208.82 (±4223.67)^B^	2924.92 (±899.83)^C^	0.001

*Different letters (^A,B,C^) denote significant differences. The same letters (^AA^) mean there are no significant inter-group differences. See the section “Methods” for task abbreviations.*

The AC group had a significantly higher mean number of correct responses for all tasks (*p*_s_ < 0.001), when compared to the DX and RC groups. The same differences were observed in relation to mean task completion time (*p*_s_< 0.001), showing that AC group participants responded faster than the other two groups.

A comparative analysis of the RC and DX groups found no significant differences in mean number of correct responses on the RCT (*p* > 0.20) and on the WSCT (*p* > 0.15). However, significant differences were observed in the mean number of items correctly responded on the PPWT, written test (*p* < 0.007), on which the RC group scored higher. Note that since the WSCT was used to match the DX with the RC group, the two were not compared on this task.

When comparing groups on mean test completion times, results indicated a significant difference on both the RCT (*p* = 0.007) and WSCT (*p* = 0.012), with the DX group taking longer to complete both tasks. The DX group took about 2 s longer than the RC and 6 s longer than the AC to judge whether an item was written correctly. However, there was no significant differences between groups on mean completion time for the OSCT (*p* = 0.845), since their times depend on the duration of the pre-recorded spoken words.

### RCT READING PATTERN

**Table 2** describes the response patterns of the three groups on the seven subitems of the RCT.

**Table 2:** Mean number of correct responses and standard deviation (SD) of the three groups on the seven subitems of the RCT.

	DX	RC	AC	
	
RCT item	Mean (SD)	Mean (SD)	Mean (SD)	Summary
CR	**9.32 (±0.82)**^a^	**8.07 (±2.34)**^c^	9.42 (±0.64)	RC < DX < AC
CI	8.54 (±1.45)	**7.61 (±2.48)**^c^	9.23 (±0.59)	RC < AC
PE	9.21 (±1.20)	9.11 (±1.23)	9.73 (±0.60)	RC = DX = AC
TS	9.61 (±0.69)	9.32 (±1.12)	9.65 (±0.56)	RC = DX = AC
TV	**7.18 (±1.96)**^A,b^	**8.11 (±1.69)**^c^	8.96 (±1.22)	Dx < RC < AC
TF	**4.86 (±2.32)**^a,b^	**7.25 (±2.24)**^c^	8.58 (±1.24)	Dx < RC < AC
PH	**4.36 (±2.38)**^a,b^	**5.71 (±2.32)**^c^	8.73 (±1.59)	Dx < RC < AC

*^a^Significant differences between DX and RC groups (*p* ≤ 0.05).*

*^A^Trend (*p* = 0.07) between DX and RC groups.*

*^b^Significant differences between DX and AC groups (*p* ≤ 0.05).*

*^c^Significant differences between RC and AC groups (*p* ≤ 0.05).*

*See the section “Materials and Methods” for task abbreviations.*

Response styles of the three groups show that DX scored lower than the AC group on all RCT subitems, with significant differences for visual confusion (VC, *p* = 0.001), phonological confusion (PhC, *p* < 0.0001) and homophone pseudoword (PNw, *p* < 0.0001) items, which are related to phonological skills. There was neither significant differences in reading regular and irregular correct words items (RC and IC, *p* = 0.803 and *p* = 0.102, respectively), nor on semantic confusion (SC) items (*p* = 1.00), or on pseudowords not derived from real words (WNw, *p* = 0.074).

When children with dyslexia were compared to the RC group, they performed significantly more poorly on phonological subitems for phonological confusion (*p* = 0.000) and homophone pseudowords (*p* = 0.054). In contrast, in the regular correct words item, DX scored significantly better (*p* = 0.021) than younger reading controls. On items of irregular correct words, pseudowords not derived from real words, and semantic confusion subitems, there were no differences between these two groups (*p*_s_ > 0.05), and both got high numbers of correct responses.

## DISCUSSION

Reading and writing results, assessed by the BALE, showed significant differences between the individuals with dyslexia and the two control groups. When children with dyslexia were compared to controls of the same age, they performed poorly on all tasks showing a lower mean number of correct responses and longer mean completion times, as shown in previous studies using this same battery (Nikaedo et al., 2007; Lukasova et al., 2008). These results also support theories suggesting that individuals with dyslexia behave similarly to younger children (Bryant and Impey, 1986; Manis et al., 1996; Stanovich et al., 1997), since they achieved lower scores on all reading and writing tests and took longer to complete tasks.

By including a third group of younger readers, we were able to better investigate the statement above. Results indicated that although students with dyslexia presented similar patterns to younger children, there were important differences between these groups. While analyzing total scores of word and pseudoword reading on the RCT and oral-sentence comprehension on the OSCT, children with dyslexia showed a delay in their performance, with similar results to that of younger controls. However, significant differences in written production (PPWT), pseudoword decoding and mean completion time for word and sentence reading tasks showed that individuals with dyslexia continued to score lower, even compared with younger controls. These differences show that despite reading and writing overall decoding being similar, the dyslexia group still required more time to complete the task, thus, to decode. This is probably because reading is multiprocessual skill, therefore both groups used different decoding strategies in the tasks (Santos and Navas, 2002; Capovilla et al., 2006). In other words, while the reading control group seemed to benefit from lexical processes and moved toward a faster, automated reading, the students with dyslexia showed greater difficulties in this direct access, and used less refined processors that prevail in early stages, when learning to read and write. Therefore, children with dyslexia still do not seem to have automated direct word retrieval, as observed in lexical and semantic spelling processes. Due to their low fluency, they require additional attention resources to enable a grapheme–phoneme decoding, thus overloading phonological processes. Consequently, their reading and writing was slow and impaired even when compared with younger readers. (Cervera-Mérida and Ygual-Fernández, 2006).

Adding to this discussion, Lukasova et al. (2008) using the BALE tasks found that dyslexic children may perform well if given sufficient time to complete reading tasks, even if they have not developed effective decoding strategies. Therefore, the competence may be subdivided into reading accuracy, which is evaluated by the number of items read correctly and reading efficiency, which is measured by tracking time spent on reading.

This was also observed in the comparative analysis of students with dyslexia and age-matched controls on the word and pseudoword reading tests (RCT). Results confirmed that there were no significant differences between groups when reading regular and irregular words (RC and IC, respectively), pseudowords (WNw), or semantic confusion (SC) items. No differences for these items were also found when children with dyslexia were compared to controls matched by reading level, with exception of regular correct words (RC), on which individuals with dyslexia scored significantly higher than these younger reading controls. Children with dyslexia and age controls both obtained high levels of correct responses on the regular words items. This corroborates findings of a study conducted by Capovilla et al. (2006), who argued that regular correct words (RC) are low-complexity items, associated with short, transparent words of high-familiarity and high-frequency, thus can be decoded using more than one reading strategy (i.e., logographic, alphabetic, or orthographic). Also worth noting is that the reading controls’ significantly lower scores for this item seem to be related to their shorter experience of reading. Griffiths and Snowling (2002) reinforced this finding and emphasized that exposure to print is a highly influential factor for competent readers.

The high rates of correct responses on semantic confusion items or pseudowords not derived from real words (SC and Wnw, respectively) across all groups, relate to the item’s low level of complexity, since the written-stimulus showed no visual (orthographic) or phonological similarity with the superimposed target-picture. Due to the high level of visual contrast between printed word and target picture (e.g., the print “*train*” paired to the image of a *bus*, in an SC item; or the pseudoword “*mitu*” paired to the image of a e*yeglass,* in a Wnw item), mistakes may be easily spotted from their holsitic/logographic visual patterns, which does not require highly refined reading. So an item may be identified as an error by merely seeing that a word’s initial letter does not correspond to the picture presented (Capovilla and Capovilla, 2002; Capovilla et al., 2004; Orsati et al., 2005).

The analysis of visual confusion, phonological confusion, and pseudowords with orthographic errors but correct phoneme–grapheme conversion items (VC, PhC, and PNw, respectively) demonstrated that students with dyslexia scored significantly below *both* control groups and even lower than the younger reading controls. Given this finding, it is clear that although both groups (DX and RC) presented similar overall reading levels, the dyslexic group had more difficulty in identifying subtle changes in visual and phonological confusion (VC and PhC) items, as well as in detecting orthographic errors on the homophone pseudoword (PNw) items, involving graphic-phonemic decoding and direct access to the orthographic lexicon (Capovilla and Capovilla, 2002).

A detailed analysis of the dyslexics’ profiles in the seven subitems of the RDT show that the high rates of correct responses in regular correct and irregular correct words (CR and CI) reflect a pattern of holistic decoding of these stimuli, as well as ability to identify visual patterns of familiar, previously seen words. As mentioned above, this probably relates to the experience of having been exposed to high-frequency Portuguese words that involve holistic reading and do not necessarily imply refined graphic–phonemic decoding (Teixeira et al., 2010). This is confirmed while analyzing the performance of the group with dyslexia on phonological and visual confusion (PhC and VC) items. Their low scores on these items show that the dyslexic group was not yet able to detect subtle features that required more refined visual and phonological decoding. Also there appears to be difficulty retrieving the orthographic lexicon, since the dyslexic group was unable to recognize target-word alterations by direct visual processing, which involves previous knowledge of orthographic rules. If their lexical processing were adequate, subjects from the dyslexia group would identify errors and reject this type of item. However, it is also important to note that errors in visual confusion (VC) items may reflect phonological or central auditory processing difficulties, as stated in previous studies (Capovilla and Capovilla, 2002).

These findings are similar to those reported by Capovilla et al. (2004), in a study comparing students with dyslexia and competent readers paired by age in the RCT. Results concluded that dyslexic children had great difficulty with phonological processing and continued to use a primarily logographic style of reading, finding it hard to master alphabetic reading and then progress to orthographic reading. This pattern is consistent with proposals drawn by cognitive psychology and neuropsychology, which state that cognitive systems operate through relatively independent modules (Luria, 1981; Salles et al., 2004). In this study, predominantly phonological processes were affected, corroborating to the phonological-deficit explanatory model (Frith, 1997; Mody, 2003), which suggests that poorly developed phonological processing impacts the alphabetical route, interfering with the reader’s ability to build up an orthographic lexicon that will subsequently enable them to use orthographic rules for reading. In the case of dyslexia, therefore, deficit in the access to the mental orthographic lexicon may also arise from primary phonological difficulties (Capovilla and Capovilla, 2002; Teixeira et al., 2010).

Summing up, findings of the present study confirm that initial differences observed between children with dyslexia and age-matched controls were more clearly evidenced when a second control group, matched by reading level was introduced (Salles et al., 2004; Affonso et al., 2011). By including a wider number of tasks from the BALE, we were able to identify subtle differences in different aspects of reading and writing.

Going further, as previously mentioned, our analysis of the three groups also supports the hypothesis that the acquisition of reading and writing skills in individuals with dyslexia is delayed and similar to that of younger children (Bryant and Impey, 1986; Manis et al., 1996; Stanovich et al., 1997). However, despite this similarity, the present study confirmed that individuals with dyslexia present specific deficits that differentiate them from the latter. Longer completion times for reading tasks and more severe difficulties to process the reading of pseudowords were persistent symptoms that differentiated both groups. These findings corroborate previous studies (Shaywitz and Shaywitz, 2005) that highlighted these persistent characteristics, related to the phonological deficit hypothesis. The clear difficulty in phonological processing, thus shows that dyslexia is a particular condition that subsequently affects reading and writing skills (Frith, 1997; Mody, 2003; Salles and Parente, 2006).

Despite the similarity with younger readers, one important finding of the present study is that the persistence of some signs shows that individuals with dyslexia are affected by the development of specific reading-related skills being *diverted* rather than *delayed,* hence showing consistent differences between these two groups (Bryant and Impey, 1986; Manis et al., 1996; Frith, 1997, Ramus et al., 2003).

Another important point is that research using the BALE assessment in a Brazilian cultural context demonstrates the need of developing specific instruments for ones written language rather than adapting those used in other countries. This allows us to consider and balance important psycholinguistic aspects of the language, giving rise to a more precise profile of the child’s reading and writing development. As observed in the study conducted by Teixeira et al. (2010), when loose qualitative parameters are used to access written language, misguiding results emerge, giving wrong impressions on how students are performing in these abilities. By developing national instruments with precise paradigms, to be used not only by clinicians, but also by school teachers, we will enlarge the number of Brazilian students who are scanned as from the start of their literacy process. Hopefully this will allow us to track the long-term development of this process and as a consequence, raise the possibility of early identifying children at risk for dyslexia.

## Conflict of Interest Statement

The authors declare that the research was conducted in the absence of any commercial or financial relationships that could be construed as a potential conflict of interest.
